# Improving medication adherence with adjuvant aromatase inhibitor in women with breast cancer: study protocol of a randomised controlled trial to evaluate the effect of short message service (SMS) reminder

**DOI:** 10.1186/s12885-018-4660-7

**Published:** 2018-07-09

**Authors:** Yunxin He, Eng Hooi Tan, Andrea Li Ann Wong, Chuan Chien Tan, Patrick Wong, Soo Chin Lee, Bee Choo Tai

**Affiliations:** 1 Saw Swee Hock School of Public Health, National University of Singapore and National University Health System, 12 Science Drive 2, #10-03F, Singapore, 117549 Singapore; 2 Department of Haematology-Oncology, National University Cancer Institute, NUHS Tower Block Level 7, 1E Kent Ridge Road, Singapore, 119228 Singapore; 3 Department of General Surgery, Ng Teng Fong General Hospital, 1 Jurong East Street 21, Singapore, 609606 Singapore; 4 Division of Oncology Pharmacy, National University Cancer Institute, NUHS Tower Block Level 7, 1E Kent Ridge Road, Singapore, 119228 Singapore; 5 Yong Loo Lin School of Medicine, National University of Singapore and National University Health System, 1E Kent Ridge Road, Singapore, 119228 Singapore

**Keywords:** Randomised controlled trial, Aromatase inhibitor therapy, Breast cancer, Medication adherence, SMS reminder

## Abstract

**Background:**

Medication adherence refers to whether a patient takes medication according to the frequency prescribed, or continues to take a prescribed medication. Inadequate adherence to medication may cause alterations in risk-benefit ratios, resulting in reduced benefits, increased risks or both, and is significantly associated with adverse clinical outcomes and higher healthcare costs. We aim to examine the effect of a computer generated short message service (SMS) reminder in improving medication adherence, and inhibiting the aromatisation process amongst breast cancer women receiving oral aromatase inhibitor therapy.

**Methods/Design:**

In this randomised controlled trial, eligible patients will be equally allocated to receive either SMS reminder or standard care. The former receives weekly SMS reminder to take medication while the latter does not receive any. The primary endpoint of medication adherence at 1-year is assessed using the Simplified Medication Adherence Questionnaire, and compared using the χ^2^ test. Adjustment for baseline covariate and potential confounders will be made using the logistic regression. Secondary outcomes involving estrone and androstenedione levels will be compared using the analysis of covariance, whereas the estradiol levels (< 18.4 pmol/L versus ≥18.4 pmol/L) will be compared using the χ^2^ test, and the logistic regression. Further, the assessment of knowledge, attitude, behaviour, and barriers and facilitating factors of medication adherence will be made via logistic regression.

**Discussion:**

This will be the first study to evaluate short-term clinical outcomes from SMS reminder for breast cancer patients on aromatase inhibitor therapy. Random allocation to SMS reminder or control arm ensures that patients in both arms will be comparable with respect to demographic and clinical characteristics, and any difference in outcomes can be attributed to the intervention. Participants are not blinded to the assignment of intervention, thus there may be potential for bias in outcome assessments.

**Trial registration:**

NCT02524548. Retrospectively registered on 17 August 2015.

## Background

Medication adherence typically refers to whether a patient takes medication according to the frequency prescribed, or continues to take a prescribed medication [1]. The World Health Organisation defines medication adherence to long-term therapy as “the extent to which a person’s behaviour - taking medication, following a diet, and/or executing lifestyle changes, corresponds with agreed recommendations from a health care provider” [2]. Accruing evidences show that inadequate medication compliance can cause alterations in benefit/risk ratios, resulting in reduced benefits, increased risks or both [3, 4]. Medication non-adherence is significantly associated with adverse clinical outcomes and higher healthcare costs [5].

### Aromatase inhibitor and breast cancer risk and recurrence

Breast cancer is the leading type of cancer affecting women, and it was estimated that worldwide over 508,000 women died of this condition in 2011 [6]. In Singapore, it is also the primary cause of death for women from 2011 to 2015 [7]. Besides, a total of 9634 new cases of breast cancer were diagnosed in the same period, accounting for 29.1% of incident cancers in females and making it the most common cancer among women [7].

Estrogen promotes the growth and survival of normal and cancerous breast epithelial cells by binding and activating the estrogen receptor (ER). The activated receptor in turn binds to gene promoters in the nucleus and activates many other genes responsible for cell division, inhibition of cell death, new blood vessel formation and protease activity [8]. Thus, hormone therapy, which is a form of systemic therapy, is recommended for women with hormone receptor-positive breast cancer. Aromatase inhibitor (AI), a commonly prescribed hormonal therapy for early stage breast cancer, interferes with the body’s ability to produce estrogen from androgens by suppressing the aromatase enzyme activity. Research has shown that women treated with adjuvant hormone therapy for early-stage ER positive breast cancer gain at least 5 more years due to the treatment. However, it has been reported that patients could suffer from serious side effects of the hormone therapy [9].

Key et al. demonstrated a significant association between breast cancer risk and circulating estrogens in postmenopausal women [10]. Estrogen is the main stimulant in the development and growth of breast cancer, [11] and higher endogenous estrogen level has been found to be associated with elevated breast cancer risk. Tamoxifen has been successfully used for treating ER positive breast cancer for the past three decades. More recently, AI has been demonstrated to provide a significant reduction in breast cancer recurrence in post-menopausal women [4]. Following menopause, aromatase in fat and muscle may be responsible for much of the circulating estrogen. In highly estrogen-sensitive tissues such as the breast, uterus, vagina, bone, brain, heart and blood vessels, it provides local estrogen in an autocrine fashion. The aromatase gene promoter in breast tissue is less sensitive than the gene promoter in the ovary due to fluctuations in luteinising hormone but much more sensitive to increases in inflammatory cytokines, which increases with age. In particular, breast tissue inflammatory cytokines increase with proliferative breast disease and breast cancer [8].

A prospective, multi-centre, non-interventional study reported significant improvements in long-term quality of life of postmenopausal Chinese patients with hormone-receptor-positive early-stage breast cancer after starting treatment with AI [12]. A recent meta-analysis also showed the superiority of AI as an adjuvant hormonal therapy in improving the disease-free and overall survival of postmenopausal ER-positive breast cancer as compared to tamoxifen [13]. In another clinical trial, researchers have suggested that improvement in overall survival (hazard ratio 0.78; 95% CI 0.62 to 0.98) was seen among premenopausal women with the use of exemestane plus ovarian suppression as compared to tamoxifen users [14].

However, adverse events include hot flushes, vaginal dryness, loss of libido, fatigue, arthralgia, joint stiffness and loss of bone mineral density with subsequent increased risk of fracture [8, 15]. Also, as the treatment of AI is long-term, medication adherence is an issue of concern, with a proportion of women stopping before completing the full treatment, while others not taking a daily tablet regularly [16, 17]. Its adherence rate has been shown to decline over time from 78 to 86% in the first year, and reaching 62 to 79% by Year 3 [17]. A recent nested case-control study showed that non-adherence to oral endocrine therapy was significantly associated with worse breast cancer survival (OR 4.07; 95% CI 3.27–5.06) [18].

### Intervention to improve medication adherence

Recent reviews have shown that although various behavioural, educational, integrated care and self-management risk communication interventions have been implemented to improve medication adherence, none of them have shown promising impact [19, 20]. On the other hand, studies have suggested that reminder of any form, such as setting an alarm on a regular daily basis at home, or have family reminding the patient to take medication, have a positive influence on medication adherence in cancer patients [19]. While healthcare institutions sometimes use short message service (SMS) to remind patients of follow-up clinic appointments, mobile technology is seldom implemented to monitor medication adherence until the recent decade [21]. This is a feasible and acceptable form of managing medication which has a high participant satisfaction [22]. Its use has been shown to improve medication adherence in cardiovascular disease, diabetes, HIV, oral contraception, asthma, as well as oncology patients with chronic conditions [22, 23]. As mobile phone ownership continues to increase, there is a great potential to utilise this technology to overcome adherence barriers and optimise therapeutic effects [24].

### Barriers and facilitating factors influencing medication adherence

Malcolm et al. identified cost, side-effects, transportation, lack of reimbursement for the medication and inefficient patient-physician communication as barriers to medication adherence [25]. Medication-taking behaviour has been shown to be influenced by a patient’s belief and his/her trust in the physician who prescribes the medication [26]. Also, non-adherence to endocrine therapy might be due to patients’ response to the AI therapy and its associated side effects such as arthralgia [27]. Besides, it has been shown that nonadherence to medications for chronic diseases prior to hormonal therapy was associated with more severe nonadherence to oral hormonal therapy in patients with breast cancer [28] A cross-sectional study comprising mostly White women showed that survivors who perceived that their pain made taking AIs difficult or that the AI treatment lasted too long were likely to be non-adherent, as assessed by the Health Beliefs Scale Items and Scale Properties [29]. In contrast, a systematic review showed that taking more medications at baseline, referral to an oncologist, and being diagnosed at earlier times were positively associated with adherence and/or persistence [16]. Thus, in non-adherent patients, it is desirable to understand their attitude and perception towards medication taking.

Cancer treatment adherence plays a crucial role in optimising health outcomes while medication non-adherence is associated with decreased survival, higher recurrences and increased healthcare costs. Hsieh et al. reported that interruption and non-adherence to long-term adjuvant hormone therapy was associated with increased mortality in breast cancer women [30]. Treatment non-adherence to adjuvant hormonal therapy was found to be associated with increased all-cause mortality amongst Asian breast cancer women. Moreover, a greater impact of non-adherence on mortality was especially found in the younger breast cancer population [30].

Implementing innovative technology to tackle the problem of non-adherence and understanding its barriers will provide scientific evidence for clinical decision making and improve health outcomes. We thus propose to examine the effect of a computer automated SMS reminder in improving medication adherence amongst breast cancer women receiving oral AI therapy.

### Study objectives

The primary objective is to evaluate whether SMS reminder improves medication adherence as compared to standard care (control) amongst breast cancer women who have been receiving adjuvant endocrine therapy for at least 1 year, and are continuing to receive adjuvant AI therapy for at least 1 more year.

The secondary objectives are to examine whether SMS reminder improves the inhibition of aromatisation process of patients receiving AI therapy. We postulate that at 1-year, there will be

- Lower estrone and estradiol levels in the SMS reminder group as compared to the control.
- Higher androstenedione level in the SMS reminder group as compared to the control.

Tertiary objectives include

- Assessing the impact of SMS reminder on the improvement in knowledge, attitude and behaviour towards medication compliance.
- Identifying barriers and facilitating factors for medication adherence.

## Methods/Design

### Study design

This is an open-label, multi-centre prospective randomised controlled trial of SMS reminder versus standard care to investigate whether SMS reminder improves adherence to oral AI therapy in breast cancer women at 1-year follow-up. A total of 280 breast cancer women will be recruited from the National University Cancer Institute, Singapore and the Ng Teng Fong General Hospital, Singapore. Eligible participants will receive financial compensation for each study visit in an amount within the guidelines of the ethics review board.

### Inclusion criteria

- Women who have been receiving adjuvant endocrine therapy for at least 1 year, and are continuing to receive adjuvant AI therapy for at least 1 more year.
- Aged 21 to 80 years.
- Have cellular phone that can receive text messages.
- Singaporean or permanent resident who is currently residing in Singapore.
- Able to give informed consent.

### Exclusion criteria

- Unable or not willing to comply with study procedures.

### Study intervention

**Fig. 1:**
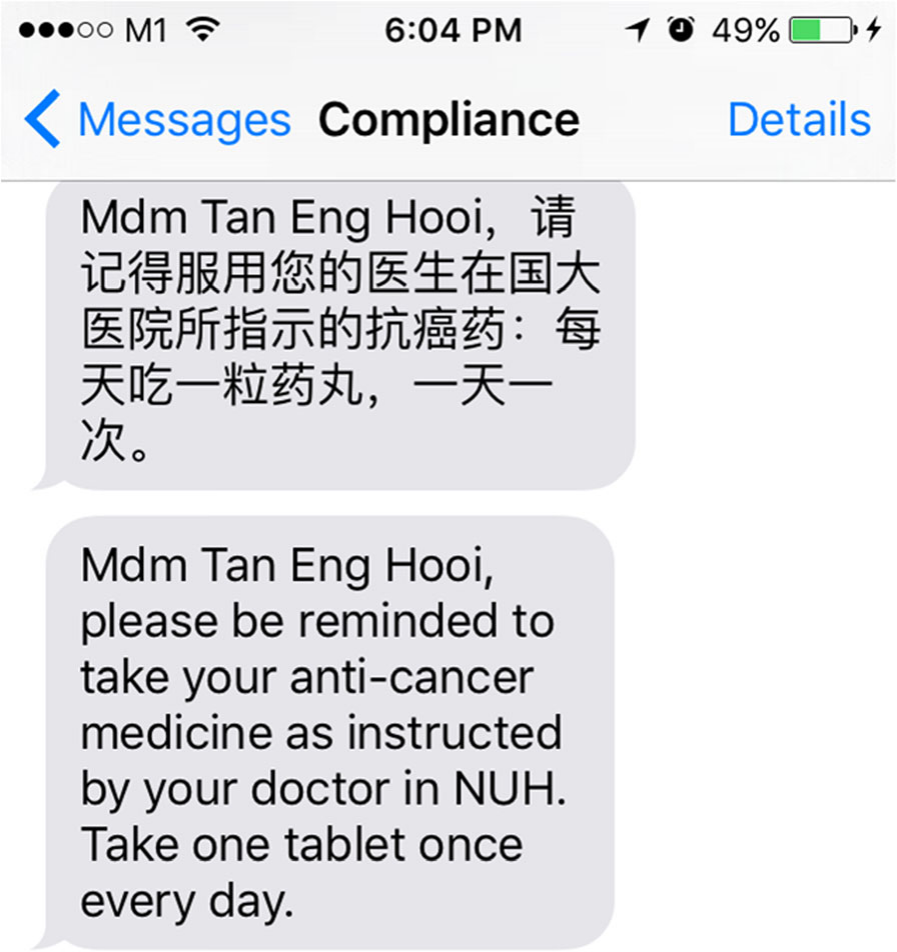
SMS reminder from the automated messaging system

A systematic review involving HIV patients found weekly reminders to significantly improve the percentage of achieving 90% adherence, while daily reminders showed no improvement [31]. Thus, we propose to send weekly SMS reminders on the first working day of each week, that is, at 9:00 AM each Monday morning from a tablet via an automated messaging system, throughout the 1-year follow-up period for the intervention group. A screen shot of the message sent is shown in Fig. 1.

The message includes information on patient’s name, date, hospital, medication and frequency of oral AI therapy. In order to cater to the multi-ethnic study population, the message is translated to Mandarin or Malay. If a patient is not proficient in English, the messages will be sent using her preferred language. The control group will not receive any message. Regardless of the randomised allocation, all patients will be given a log for the next 6 months to record whether they have received the message (for the intervention group) or taken the pills according to instruction. The log will be recalled on the subsequent visit and a new log will be distributed to patients for recording the information for the subsequent 6 months. This log will be returned to the study coordinator at the end of the study.

### Randomisation procedure

**Fig. 2:**
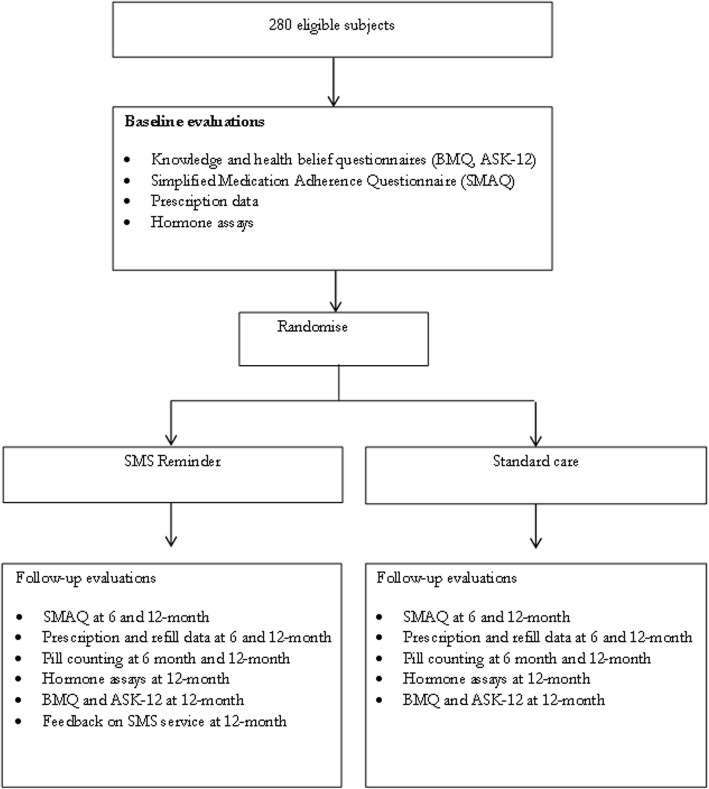
Study flow chart

All eligible patients who have provided informed consent will be randomised to receive either SMS reminder or standard care in a 1:1 ratio. Balanced permuted block randomisation will be implemented with varying block sizes, stratified by centre. Allocation concealment will be ensured, as the randomisation allocation, generated by the responsible statistician, will not be released to the research coordinator until the patient has been recruited into the trial by means of a central telephone call upon meeting the eligibility criteria. The study flow chart is shown in Fig. 2.

### Data collection

Three questionnaires will be administered via face-to-face interview by the Research coordinator. The Simplified Medication Adherence Questionnaire (SMAQ) is a validated 6-item questionnaire which measures adherence [32]. Non-compliance is defined if a patient responds to any of items 1 to 4 with a non-adherence answer; and if the patient has skipped more than two doses during the last week or has not taken medication for more than two complete days during the last visit. In addition, information on knowledge, attitude and behaviour towards medication taking will be elicited via the Beliefs about Medicines Questionnaire (BMQ) [33] and the Adherence Starts with Knowledge (ASK-12) questionnaire [34].

### Follow-up and assessments

The assessments and follow-up schedule (with a window period of ±2 months) are summarised in Table 1.

**Table 1:** Summary of study procedures and schedule

Study procedures	Visit 1	Visit 2	Visit 3
	Screening/Baseline	Month 6	Month 12
Informed consent	x		
Assess eligibility	x		
SMAQ	x	x	x
BMQ, ASK-12	x		x
Prescription data	x	x	x
Pill count and refill data		x	x
Estrone, estradiol and androstenedione assays	x		x
UPIRTSO event		x	x
Patient log of daily pill taken and weekly SMS		x	x
^a^Check receipt of SMS	x	x	x

^a^Research coordinator will verbally check the status of SMS receipt at each clinic visit

The patients will be followed up half yearly. The SMAQ will be administered at each visit, while the BMQ and ASK-12 questionnaires will be administered at baseline and 1-year. The hormone assays of estrone, estradiol and androstenedione will also be performed at baseline and 1-year.

This trial does not involve any experimental treatment and thus poses no additional risks to patients. Nevertheless, at each follow-up visit, the research coordinator will record any symptom or event that a patient may have experienced that is considered as UPIRTSO (Unanticipated Problems Involving Risks to Subjects and Others) event. These events will be monitored closely using a log book.

### Statistical considerations

#### Sample size

The sample size is estimated based on the primary endpoint of medication adherence (as measured via SMAQ) at 1-year. Assuming that the proportion of medication adherence in the intervention and control groups are 80 and 60% respectively, [17] then based on a 5% significance level and a power of 90%, a minimum sample of 240 subjects (i.e. 120 per group) will be required. Further assuming an attrition rate of 10%, the overall trial size is 280.

#### Statistical analysis plan

The assessment of medication adherence at 1-year between the SMS reminder and control groups will be made using the χ^2^ test, with adjustment for potential confounders made using the logistic regression.

Natural log transformation will be implemented on the estrone and androstenedione measures to normalise the data. The comparison of secondary outcomes involving estrone and androstenedione levels will be made using the analysis of covariance (ANCOVA) to adjust for the respective baseline levels and other potential confounders. The estradiol levels (defined as < 18.4 pmol/L versus ≥18.4 pmol/L) will be compared between the two arms using the χ^2^ test and the logistic regression analysis will be implemented to adjust for baseline covariate and other potential confounders where appropriate.

The assessment of knowledge, attitude, behaviour, as well as barrier and facilitating factors between the intervention and control groups will be made using χ^2^ tests, with treatment effect quantified based on odds ratio estimate and its 95% confidence interval. Further adjustment for baseline scores and other potential confounders will be made using the logistic regression analysis where appropriate.

All analyses will be performed according to the principle of intention-to-treat using STATA version 15, assuming a two-sided test at the 5% level of significance.

### Assessing medication adherence

There are different ways of assessing medication compliance. Patient self-reporting relies mainly on a patient’s recall, and is susceptible to bias. Pill count has also been shown to be unreliable especially if patients fail to return the bottles or dumped the pills before the count [35]. Medication possession ratio is commonly used for claims-based adherence measurement. However, it overestimates the true adherence rate particularly when a patient receives an early refill of the medication. The proportion of days covered (PDC), a recent method for calculating adherence, avoids double counting when refills overlap or when oversupply of medication exists [36]. However, it ignores instances when patients refill their prescriptions before finishing the drug.

In a further sub-study, we will compare the four different measures of adherence, to determine which of these provide a more meaningful or reliable information in the local context based on different performance indicators such as the sensitivity, specificity and kappa statistics using PDC as the gold standard. The percentages of adherence between the different measures will be compared via the McNemar’s test.

### Ethics and dissemination

Informed consent will be obtained by the co-Principal Investigators or research coordinator during the screening visit, prior to randomisation into the trial. The patient’s consent to participate in the trial should be obtained after a full explanation has been given of the intervention options and the manner of intervention allocation. A copy of the signed consent form with study information will be given to the patients for their retention. The right of the patient to refuse to participate without giving reasons must be respected. After the patient has entered the trial, the clinician must remain free to give alternative intervention to that specified in the protocol at any stage if she feels it to be in the patient’s best interest. The patient must remain free to withdraw at any time from the protocol intervention without giving reasons and without prejudicing her further treatment.

The protocol and the associated informed consent documents have been reviewed and approved by the institutional review board of both study sites. The results of this trial will be published in a peer-reviewed journal.

### Data quality assurance

To ensure accuracy, completeness and reliability, the data will be prospectively collected and entered into the electronic database. Hardcopies of the data collection forms will be checked for completeness and verified by the research coordinator before data entry. Validity range checks will also be built into the database. At the study closure, 10% of the records will be randomly sampled for data quality assurance.

### Confidentiality of data and patient records

All data will be treated with strict confidentiality and will not be shared with parties other than members of the investigating team. All data will be centrally coordinated and the hardcopy forms will be kept under lock-and-key in a cabinet in the research coordinator’s office. The softcopy data will be stored in a hard disk and protected by password. Subjects will be identified by a unique trial number and their identities will not be revealed in the data collection forms or questionnaires. A patient study ID list containing patient’s name, personal identification number, mobile phone number and trial number will be maintained and kept in a study folder separate from the data collection forms.

## Discussion

In recent years, various interventions have been utilised to improve medication adherence to AI therapy for breast or other types of cancer [37, 38]. A pilot randomised controlled trial of a web-enabled application to provide real-time monitoring and better management of treatment-related adverse symptoms among patients with hormone-receptor positive breast cancer showed web-enabled application with weekly reminders significantly improved short-term AI adherence. Also, several studies have suggested that short message reminder could benefit adherence to breast self-examination or cancer-screening among breast cancer patients [39, 40]. Chung et al. suggested that self-reported breast self-examination (BSE) adherence and the frequency of BSE were significantly higher in the intervention group than in the control group in the 6-month period [39]. However, to our knowledge, there has not been any study involving short-term clinical outcomes such as hormonal assays from reminders for breast cancer patients on AI therapies.

Studies on breast cancer have demonstrated that patients commonly reported suffering from physical and psychological inconvenience due to AI therapies [41]. As compared to women without a history of cancer, breast cancer patients, reported significantly higher odds of new onset of forgetfulness, difficulty in concentrating, hair loss and numbness or tingling in the extremities after the first 6 months of AI therapy, with odds ratio ranging between 2 to 4 folds [41]. A German study also showed that age contributed to the difference in medication adherence amongst breast cancer women who were treated with AI [42]. It suggested that patients aged over 70 years exhibited a 25% reduction in risk of treatment discontinuation as compared to younger patients. However, a literature review discussing poor adherence with hormonal therapy among women with breast cancer disagreed with the notion that non-adherence could be due to older age (≥55 years). It reported that other reasons for hormonal therapy non-adherence could be due to patient’s behaviour, treatment toxicity, cost of health care or medications, and comorbidities [43]. Also, it has been further suggested that medication compliance may be improved if financial barriers were removed [43]. Meanwhile, other researchers have identified socio-demographic and clinical characteristics to be associated with medication beliefs about AI such as concern, perceived necessity of taking the drug to improve one’s health, and cancer/health worry amongst postmenopausal women [44].

This trial has several strengths as well as limitations. This will be the first study to evaluate short-term clinical outcomes from SMS reminder for breast cancer patients on AI therapy. Random allocation to SMS reminder or control arm ensures that patients in both arms will be comparable with respect to demographic and clinical characteristics, and any difference in outcomes can be attributed to the intervention. However, participants are not blinded to the assignment of intervention, thus there may be potential for bias in outcome assessments.

In short, sending SMS reminder to early-stage breast cancer women could be an added feature to existing healthcare protocol for breast cancer women if the results from our trial are promising. Understanding the potential barriers to medication adherence would enable policy makers to make informed decision when promoting regulations to minimise the impact of the barriers while formulating policies to elevate medication adherence.

## Abbreviations

AI: Aromatase inhibitor
ANCOVA: Analysis of covariance
ASK: Adherence Starts with Knowledge
BMQ: Beliefs about Medicines Questionnaire
BSE: Breast self-examination
ER: Estrogen receptor
NCIS: National University Cancer Institute, Singapore
PDC: Proportion of days covered
SMAQ: Simplified Medication Adherence Questionnaire
SMS: Short message service
UPIRTSO: Unanticipated problems involving risks to subjects and others
